# Comparison of Standard Electrocardiography and Smartphone-Based Electrocardiography Recorded at Two Different Anatomic Locations in Healthy Meat and Dairy Breed Does

**DOI:** 10.3389/fvets.2020.00416

**Published:** 2020-08-13

**Authors:** Joe S. Smith, Jessica L. Ward, Benjamin K. Schneider, Fauna L. Smith, Mikaela S. Mueller, Meera C. Heller

**Affiliations:** ^1^Department of Veterinary Diagnostic and Production Animal Medicine, College of Veterinary Medicine, Iowa State University, Ames, IA, United States; ^2^Department of Biomedical Sciences, College of Veterinary Medicine, Iowa State University, Ames, IA, United States; ^3^School of Veterinary Medicine, William R. Pritchard Veterinary Medical Teaching Hospital, University of California, Davis, Davis, CA, United States; ^4^Department of Veterinary Clinical Sciences, College of Veterinary Medicine, Iowa State University, Ames, IA, United States; ^5^Department of Medicine and Epidemiology, School of Veterinary Medicine, University of California, Davis, Davis, CA, United States

**Keywords:** dairy goat, ECG, ECG & wireless, goat (*Capra aegagus hircus*), meat goat, smartphone

## Abstract

Smartphones present multiple applications for ambulatory practice. One of the newer technologies is smartphone-based electrocardiography (ECG). While this technology has been explored in horses and cattle, it has not yet been evaluated for goats. Fifteen goats of dairy and meat breeds were simultaneously tested with both a standard and smartphone-based ECG from two different anatomic locations (base apex and sternal positions). ECGs were compared for quality score, heart rate, and ECG intervals. Smartphone-based ECGs were feasible to collect in all goats under field settings. Scoring indicated higher quality scores for the standard ECG when compared to the smartphone-based ECG, and differences in smartphone ECG quality scores were noted between goats of different body types. Heart rate agreement was noted between measurements taken from smartphone-based and standard devices. ECG intervals calculated for smartphone-based ECGs were clinically similar to standard ECG. While not of the same diagnostic quality as standard ECG recordings, smartphone-based ECGs for goats present an easy to collect recording for caprine practice.

## Introduction

The devices currently described for heart rhythm evaluation in the goat are standard ECG units with associated cables and electrodes (1, 2). The use of these devices for goats can be problematic, as they are not easily adapted for field use and may be economically limiting for small ruminant practice. The pairing of smartphone technology with a wireless enabled ECG recorder has resulted in the ability to record ECG tracings with less cumbersome equipment than has traditionally been utilized for veterinary species. These devices have demonstrated efficacy for cats (3), dogs (4), horses (5, 6), dairy cattle (7), and water buffalo calves (8), but currently no studies describe the use of this new technology for goats.

The smartphone-enabled ECG device allows for stall-side collection of both heart rate and an ECG tracing, making this a potential convenient diagnostic modality for ambulatory small ruminant practitioners. The goal of this study was to investigate feasibility of a smartphone-based ECG device for heart rate and rhythm assessment in healthy does under field conditions. Additional goals included comparing heart rate, ECG time intervals, and quality scores between simultaneously-recorded smartphone ECGs (base apex positioning, BA) and standard ECGs. The final goal of the project was to compare effect of anatomic recording location (BA vs. sternal placement, ST) and breed on smartphone ECG quality scores and heart rate.

## Materials and Methods

### Animals

Eight Boer does (range 1–5 years) and nine dairy breed does (5 Alpine and 4 La Mancha; range 2–6 years) weighing 70.3 ± 13.0 kg were recruited from the University of California Davis Animal Science goat teaching and research facility. All does were screened for health prior to the study by history and physical examination. None of the does were pregnant at the time of the study. The study protocol was approved by the Institutional Animal Care and Use Committee of the University of California (Protocol # 18685).

### Data Collection

Standard base-apex ECG protocol was performed as described for cattle by Bonelli et al. (7). Does were manually restrained while standing via holding of a neck collar. Standard ECGs were collected via a commercial ECG recorder (MAC-1200 ECG System, Marquette Hellige GmbH, Germany) using standard base-apex placement of the negative lead on the lower 1/3 of the left jugular furrow, the positive lead at the left 5th intercostal space caudal to the olecranon, and the third lead at the region of the point of the left shoulder as previously described for dairy cows (7). Smartphone ECG monitoring occurred simultaneously with the smartphone monitor (AliveCor Veterinary Heart Monitor, AliveCor, San Francisco, California) placed sequentially at two different locations, in random order: on the left chest wall ventral to the positive lead (base-apex placement, BA), or on the ventral midline centered on the xiphoid region (sternal placement, ST). ST was utilized as the authors observed some goats found BA placement uncomfortable during pilot testing. The smartphone device was aligned as described for dairy cattle (7) for BA placement, with the device placed on the left chest wall slightly below the olecranon and was positioned with the camera aperture aligned cranially for ST placement. After simultaneous recording was collected from one location, the alternate placement was then recorded. An iPhone 5S (Apple Inc, Cupertino, California) was used with a commercial application (AliveCor Vet, AliveCor, San Francisco, California) to record the smartphone ECGs. Hair was not clipped, and 70% isopropyl alcohol was applied to the skin for improved contact. A minimum of 15 s of ECG tracing was recorded from each device at each location. All ECGs were recorded at a paper speed of 25 mm/s and amplitude of 10 mm/mV.

### Data Analysis

After collection, all tracings were masked for subject identity and submitted to a single board-certified veterinary cardiologist (JLW) for review. Heart rate was measured manually from printed ECGs for both standard and smartphone ECGs (BA and ST); heart rate as automatically calculated via the smartphone app was also recorded. Complex measurements (amplitudes and durations of all waveforms and intervals) and assessment of cardiac rhythm were performed on standard and smartphone BA ECGs as previously reported (7, 8). Additionally, all ECGs were quality scored based on the presence or absence of baseline undulation and tremor artifacts using a three point scoring system (lowest possible = 0; highest possible = 3) previously described for veterinary use (8, 9). With this system a high quality recording with no baseline wander or small baseline deflections would be scored “0,” a recording with intermittent mild, tremors, or baseline deflection would be scored “1,” a recording with moderate tremors or consistent baseline deflection would be scored “2” and a recording with severe tremor interfering with the interpretation of P and T waves would be scored “3” (9). Using this whole number scoring system, a lower score is indicative of a higher quality ECG.

Quantitative data were assessed for normality and reported as mean, standard deviation, minimum and maximum. A heart rate in excess of 110 bpm was considered tachycardic and below 70 was considered bradycardic (10). Heart rate, and quality scores were compared between standard and smartphone ECGs as previously reported for dairy cattle (7) and water buffalo calves (8). Heart rate was manually determined by evaluating the paper ECG, as well as through the app. Paired ECGs were compared for heart rate agreement with Bland-Altman reporting of bias and 95% limits of agreement as well as Pearson's correlation. Similar analyses were performed between standard and BA smartphone ECGs for PR and QT intervals as well as QRS complex durations. Quality scores were analyzed using paired *T*-tests to compare between location (BA vs. ST) and breed (meat breed vs. dairy breed). Statistical analyses were performed with a commercial software program (Graphpad Prism 8.0.2, La Jolla, CA). A *P*-value of <0.05 was considered statistically significant.

Quality of ECG tracings taken by standard base-apex leads were evaluated against smartphone-based ECG tracings using the conditional inference procedures for testing independence as implemented in the coin package (version 1.3-1, for R 3.6.1) and detailed in Hothorn et al. (11). Ten thousands Monte Carlo samples were used to approximate the conditional null distribution. The null hypothesis tested was whether quality score (as an ordered variable) was conditioned on device and goat, when grouped under placement. A *P*-value of <0.05 was considered statistically significant.

## Results

### Data Collection and Analysis

**Figure 1:**
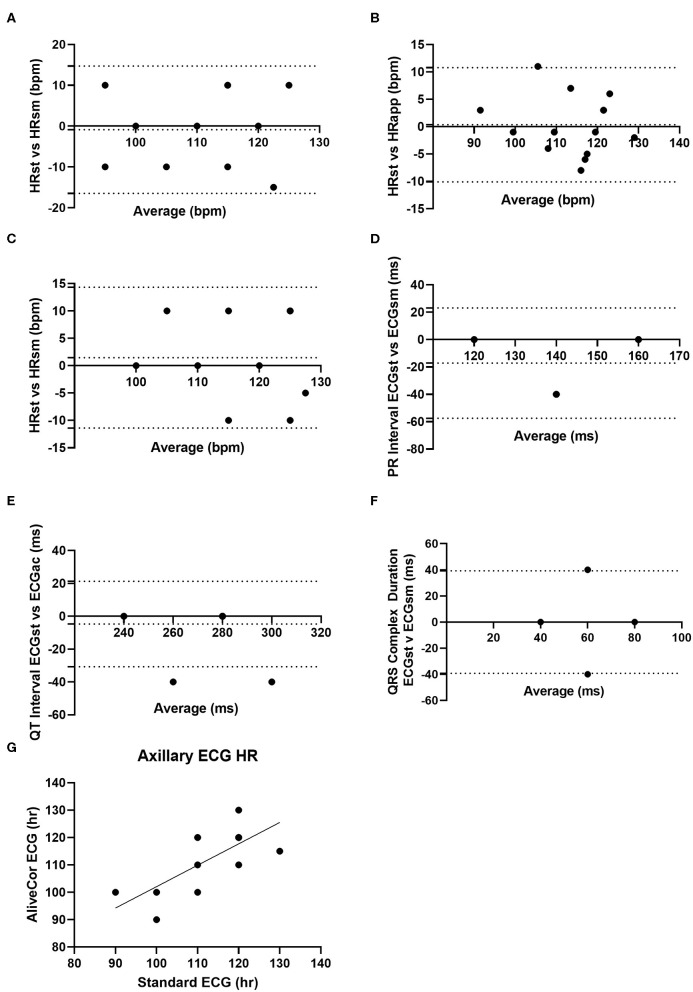
Graphical agreement between smartphone and standard ECG parameters from study goats. **(A)** Bland-Altman plot of heart rate as collected by standard ECG (HRst) and smartphone ECG (HRsm) collected from base-apex positioning. **(B)** Bland-Altman plot of heart rate collected from standard ECG (HRst) and as calculated by the AliveCor App (HRapp). **(C)** Bland-Altman plot of the heart rate as collected by standard ECG and smartphone ECG with sternal positioning. **(D)** Bland-Altman plot of the PR interval collected via standard ECG (ECGst) and smartphone (ECGsm) via base-apex positioning. **(E)** Bland-Altman plot of the QT interval as collected by standard ECG and smartphone ECG via base-apex positioning. **(F)** Bland Altman of QRS complex duration as collected by standard ECG and smartphone ECG via base-apex positioning. **(G)** Correlation of heart rate as collected from standard ECG and smartphone ECG with base-apex positioning. For Bland-Altman plots the dotted lines represent the bias and 95% limits of agreement.

ECG recording was feasible with the standard and both smartphone methods in all does (Figure 1). Seven out of 16 does moved during smartphone BA collection, and the device had to be repositioned and the recording repeated. No movement from the does was noted during data collection from the ST location. All does demonstrated normal sinus rhythm on all ECGs (standard and smartphone), with no arrhythmias noted. On standard ECG, 10 of 17 (59%) does were tachycardic and 7 of 17 (41%) had normal heart rates. No does were bradycardic.

**Figure 2:**
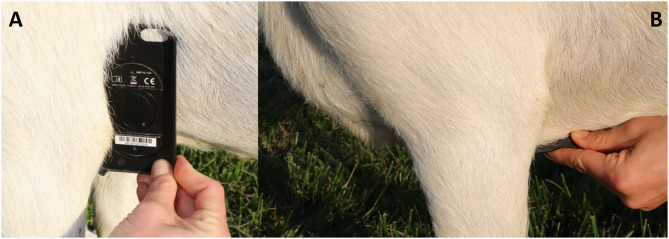
Images depicting placement of smartphone ECG device. **(A)** Represents base-apex (BA) positioning. **(B)** Represents sternal (ST) placement.

Heart rate, quality scores, and ECG interval measurements for the different ECG formats from BA placement are summarized in Table 1, while heart rate and quality scores for ECGs collected from ST placement are summarized in Table 2. When heart rate measurements were compared between standard ECG and smartphone BA, the observed bias was −0.8824 (±7.952) with 95% limits of agreement of −16.47 and 14.7 bpm. When heart rates were compared between standard ECG and smartphone ST, the observed bias was 1.471 (±6.559) with 95% limits of agreement of −11.38 and 14.44 bpm (see Figure 1). Heart rate correlation between standard and BA smartphone ECGs was moderate (*r* = 0.7134, *p* = 0.0013), and between standard and ST smartphone placement was moderate (*r* = 0.8138, *p* < 0.001) for HR values calculated manually and automatically. Figure 2 demonstrates the collection of BA and ST data with the smartphone device.

**Table 1:** Mean, standard deviation, maximum and minimum values for cardiac parameters simultaneously measured with standard electrocardiogram (ECG) and smartphone ECG recorded from a base-apex position.

**Parameter**	**Standard ECG Mean ± SD (Range)**	**Smartphone ECG Mean ± SD (Range)**
Heart Rate (bpm)	113.5 ± 10.6 (100–130)	115 ± 10.9 (100–130)
Heart Rate App (bpm)	–	113.8 ± 9.2 (93–128)
Quality Score	0.94 ± 0.56 (0–2)	1.88 ± 0.60 (1–3)
PR Interval (ms)	154.2 ± 16.5 (120–160)	135.4 ± 19.9 (120–160)
QT Interval (ms)	280 ± 17.1 (240–320)	275 ± 15.7 (240–320)
QRS Interval (ms)	75 ± 15.7 (40–80)	75 ± 15.7 (40–80)
P Amplitude (mV)	0.20 ± 0.05 (0.1–0.3)	0.05 ± 0.07 (−0.05–0.2)
QRS Amplitude (mV)	−0.39 ± 0.37 (−0.6–0.4)	−0.01 ± 0.32 (−0.6–0.4)
T Amplitude (mV)	0.33 ± 0.17 (0.15–0.75)	0.02 ± 0.14 (−0.2–0.3)

*“Heart Rate App” indicates the heart rate as automatically calculated by the AliveCor Device*.

**Table 2:** Mean, standard deviation, maximum and minimum values for heart rate and quality score simultaneously measured with standard ECG and smartphone ECG recorded from a sternal (ST) placement (ST).

**Parameter**	**Standard ECG Mean ± SD (Range)**	**Smartphone ECG ST Placement Mean ± SD (Range)**
Heart Rate (bpm)	113.75 ± 10.57 (100–130)	115.31 ± 10.90 (100–130)
Heart Rate App (bpm)	–	118.63 ± 13.81 (100–155)
Quality Score	1.06 ± 0.94 (0–3)	1.81 ± 0.73 (1–3)

**Figure 3:**
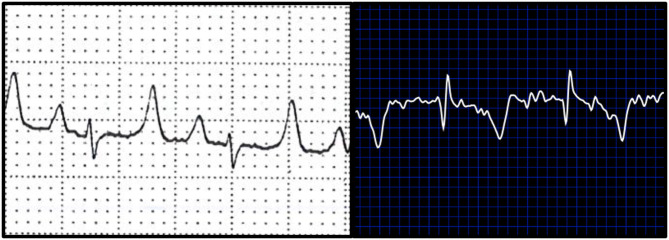
Examples of ECGs collected from a study goat. White background **(left)** represents a standard ECG. Blue background **(right)** depicts a base apex positioned smartphone ECG.

When ECG time intervals were compared against standard and BA AliveCor collected samples, agreement was noted among PR interval (bias: −17.14 ± 20.54; 95%; −57.41–23.12), QT interval (bias: −4.71 ± 13.28; 95%; −30.74–21.33), and QRS complex durations (bias: 0 ± 20.0; 95%; −39.2–39.2). PR intervals were unable to be calculated from 3 of the does from smartphone ECGs, and these were excluded from final comparisons of PR interval. When amplitudes were compared against standard ECG and smartphone BA ECGs bias (±SD), and 95% limits of agreement were: −0.1494 ± 0.0961, −0.3377–0.0391 (P); 0.3794 ± 0.4062, −0.4168–1.176 (QRS), and −0.325 ± 0.2145, −0.7454–0.0936 (T). Figure 3 is an example of a standard ECG and BA smartphone ECG of the same animal.

Quality scores were significantly better (indicated by a lower score) for standard ECG (0.94 ± 0.56) compared to BA smartphone ECG (1.88 ± 0.60; *P* < 0.0001). Quality scores were also higher for standard ECG (1.06 ± 0.94) compared to ST smartphone ECG (1.81 ± 0.73; *P* = 0.018). Quality scores did not differ between BA and ST placed smartphone ECGs (*P* = 0.7539).

Smartphone ECG quality scores were significantly lower (indicating higher quality) for meat breed does (1.53 ± 0.72) compared to dairy breed does (2.06 ± 0.56; *P* = 0.039). There was no difference in quality scores of standard ECGs between breeds (*P* = 0.2494).

Results of the conditional inference procedure indicate that (*p* < 1e^−4^) the quality scores of ECGs taken via base-apex leads were of higher quality than those of the AliveCor device.

## Discussion

This study demonstrated that smartphone-based ECG measurement was feasible in healthy does. Similar to reports in dairy cattle and water buffalo calves, smartphone ECG tracings were easy to collect under field conditions, although repositioning was necessary to record the tracing in some of the does in this study. While quality scores for smartphone ECGs were lower than those of standard ECGs, the smartphone device allowed identification of normal sinus rhythm in all goats, with clinically acceptable agreement between devices for HR and ECG time intervals collected via BA placement.

Smartphone ECG recording from a BA location yielded similar quality scores compared to the ST location, and heart rate agreement was similar amongst both smartphone placements when compared to standard ECG. Placement of the AliveCor device in locations other than the left lateral thoracic wall has been described for other species, an example being the distal thoracic limb collection from cats (3) and recording from humans where fingers from both hands are placed on the device for ECG collection (12). Either site from our study could be utilized for data collection, which may provide clinical utility for animals that are refractory to device placement from either location.

The ECGs collected during this study were collected from skin that had been prepared with alcohol for improved contact. This technique has been described for dairy cattle (7) as well as dogs (13). Practitioners should be aware that other techniques for improving skin contact have been described, such as the use of ultrasound gel or clipping of the skin (3, 8). It is currently unknown what effect, if any these methods of improving contact have on ECG quality.

An unexpected finding of this study was the differences in quality score based on body type. This could be due to breed-related differences in frame and body condition. There could be breed-based differences in some parameters in goats, similar to QT interval differences noted amongst breed in athletic horses (14). Species specific differences exist with the use of smartphone ECGs in small animal medicine, as there is frequent polarity disagreement in cats, but minimal polarity disagreement in dogs (15). The difference between meat breeds and dairy breeds may not be clinically relevant, and heart rate and rhythm were calculated successfully from both body types.

The lower quality scores of the smartphone-based ECGs in our study could have occurred for multiple reasons. All ECGs in this study were collected with does in a standing position, mimicking field settings; it is possible that motion artifacts or poor contact may have disproportionately affected smartphone-based ECG quality, since these devices contain only 2 electrodes located in close proximity. Smartphone-based ECGs may also be lower quality since electrical signals are unable to be augmented by the limbs as volume conductors, as occurs with standard ECG configuration. Despite these lower quality scores, smartphone-based ECGs in this study still demonstrated diagnostic value, similar to previous studies in large animals. In a study of horses, 48/50 smartphone ECGs were of diagnostic quality (6), this was similar to our study, with the exception of the 3 does where PR intervals were not determined. Studies of horses and dogs have found perfect heart rate agreement with smart-phone based ECGs (4, 6, 13), and while the BA heart rate agreement in the does of this study was not perfect, it was close enough that differences may be clinically insignificant (113.5 ± 10.6 vs. 115 ± 10.9 bpm). In cattle smartphone ECGs were found to be reliable indicators of heart rate and some ECG parameters (7), and this appears to be in agreement with the findings of the does in the study.

This study had several limitations. While this study was performed under realistic field conditions, animals from only one farm were used. Similarly, a small number of animals was used in our study, although efforts were made to incorporate individuals of both dairy and meat breeds. Another limitation of this study was the recording of tracings at 25 mm/sec, which may limit the sensitivity of detecting differences in waveform intervals. Smartphone-based ECG recording duration in this study was relatively short (15 s), which may have influenced accuracy of the application's algorithm for average heart rate determination. An additional limitation of this study is the use of healthy animals without underlying cardiac pathology, with all patients displaying normal sinus rhythm. Further work will be necessary to determine the applicability of smartphone-based ECGs in the diagnosis of caprine arrhythmias. In dogs, the smartphone ECG represents an additional tool in the diagnosis of arrhythmias, but is not a substitute for a 6-lead ECG (4). Additional future studies should also consider testing of additional smartphone-based ECG devices on the market, such as the Kardia (AliveCor) and the ECG check (Cardiac Designs) (16). Similarly, caution should be used when interpreting results from the smartphone device instead of a traditional ECG recorder in goats. Future work evaluating this device for feasibility of monitoring for procedures requiring sedation or anesthesia also warrants further exploration.

In conclusion, the smartphone ECG presents an opportunity for heart rate and cardiac rhythm analysis in goats in field conditions or stall-side, as supported by the evaluation of sinus rhythm. Of the two locations examined, both provided similar heart rate agreement and quality scores. While not a substitute for standard ECG collection, the smartphone device does provide a preliminary diagnostic tool for caprine practitioners for investigation of heart rate and rhythm in healthy goats.

## Data Availability Statement

All datasets generated for this study are included in the article/supplementary material.

## Ethics Statement

The animal study was reviewed and approved by Institutional Animal Care and Use Committee, University of California.

## Author Contributions

JS and MH contributed to study design, execution, data analysis, and manuscript construction. JW and BS contributed to study execution, data analysis, and manuscript construction. FS contributed to study execution. MM contributed to study design and execution. All authors contributed to the article and approved the submitted version.

## Conflict of Interest

The authors declare that the research was conducted in the absence of any commercial or financial relationships that could be construed as a potential conflict of interest.
